# The Cholinergic System Contributes to the Immunopathological Progression of Experimental Pulmonary Tuberculosis

**DOI:** 10.3389/fimmu.2020.581911

**Published:** 2021-02-18

**Authors:** Leon Islas-Weinstein, Brenda Marquina-Castillo, Dulce Mata-Espinosa, Iris S. Paredes-González, Jaime Chávez, Luciana Balboa, José Luis Marín Franco, Daniel Guerrero-Romero, Jorge Alberto Barrios-Payan, Rogelio Hernandez-Pando

**Affiliations:** ^1^ Division of Experimental Pathology, Department of Pathology, National Institute of Medical Sciences and Nutrition Salvador Zubirán, México City, Mexico; ^2^ Department of Bronchial Hyperreactivity, National Institute of Respiratory Diseases (Mexico), Mexico City, Mexico; ^3^ Laboratorio de Inmunología de Enfermedades Respiratorias, Instituto de Medicina Experimental del National Scientific and Technical Research Council (CONICET), Academia Nacional de Medicina, Buenos Aires, Argentina; ^4^ Departamento de Matemáticas, Escuela Superior de Física y Matemáticas, Instituto Politécnico Nacional, Mexico City, Mexico

**Keywords:** cholinergic, pulmonary inflammation, mycobacterium tuberculosis, acetylcholine, nAChR antagonism, immune response, tuberculosis, choline acetyltransferase

## Abstract

The cholinergic system is present in both bacteria and mammals and regulates inflammation during bacterial respiratory infections through neuronal and non-neuronal production of acetylcholine (ACh) and its receptors. However, the presence of this system during the immunopathogenesis of pulmonary tuberculosis (TB) *in vivo* and in its causative agent *Mycobacterium tuberculosis* (*Mtb*) has not been studied. Therefore, we used an experimental model of progressive pulmonary TB in BALB/c mice to quantify pulmonary ACh using high-performance liquid chromatography during the course of the disease. In addition, we performed immunohistochemistry in lung tissue to determine the cellular expression of cholinergic system components, and then administered nicotinic receptor (nAChR) antagonists to validate their effect on lung bacterial burden, inflammation, and pro-inflammatory cytokines. Finally, we subjected *Mtb* cultures to colorimetric analysis to reveal the production of ACh and the effect of ACh and nAChR antagonists on *Mtb* growth. Our results show high concentrations of ACh and expression of its synthesizing enzyme choline acetyltransferase (ChAT) during early infection in lung epithelial cells and macrophages. During late progressive TB, lung ACh upregulation was even higher and coincided with ChAT and α7 nAChR subunit expression in immune cells. Moreover, the administration of nAChR antagonists increased pro-inflammatory cytokines, reduced bacillary loads and synergized with antibiotic therapy in multidrug resistant TB. Finally, *in vitro* studies revealed that the bacteria is capable of producing nanomolar concentrations of ACh in liquid culture. In addition, the administration of ACh and nicotinic antagonists to *Mtb* cultures induced or inhibited bacterial proliferation, respectively. These results suggest that *Mtb* possesses a cholinergic system and upregulates the lung non-neuronal cholinergic system, particularly during late progressive TB. The upregulation of the cholinergic system during infection could aid both bacterial growth and immunomodulation within the lung to favor disease progression. Furthermore, the therapeutic efficacy of modulating this system suggests that it could be a target for treating the disease.

## Introduction

The cholinergic system is responsible for the coordinated synthesis, effects, and degradation of acetylcholine (ACh), an endogenous nicotinic receptor (nAChR) and muscarinic receptor (mAChR) agonist (1). Although ACh is the primary parasympathetic neurotransmitter of the airways, non-neuronal cells are crucial sources of ACh production (2). Through nAChR and mAChR stimulation, ACh modulates airway inflammation in chronic inflammatory lung disease (3, 4) and in bacterial respiratory infection (5–9). However, its dynamics during pulmonary tuberculosis (TB) remain unknown.

Visceral afferent neurons monitor and transmit information from strategically located peripheral sites associated with infection and injury, and through a reflex fashion return efferent autonomic signals to regulate localized immune responses. In the lungs, vagal sensory neurons are activated during bacterial or viral infection, cellular damage, and in airway allergenic responses (10). Cholinergic modulation of the lung’s immune response during normal conditions and in inflammatory conditions is partly mediated by the “pulmonary parasympathetic inflammatory reflex” (PPIR) (11), which is comparable to the spleen’s inflammatory reflex (12). This reflex is initiated with stimulation of C-fiber receptors, cytokine receptors and toll-like receptors in pulmonary afferent vagus nerve fibers and neural signal relay to the solitary tract nuclei. Subsequent activation of the dorsal motor nucleus causes efferent vagus nerve activation and ensuing neuronal and non-neuronal ACh release within the lung. The released ACh downregulates local leukocyte proinflammatory cytokines output through NF-κB interference (11). Importantly, microorganisms may have developed strategies to hijack the PPIR to induce pulmonary immunomodulation and infection progression (5, 7, 11, 13).

Despite the widespread use of antibiotic combination therapy, TB remains the most lethal infectious disease worldwide and many aspects of its pathogenesis remain unknown (14). ACh has not been studied in a pulmonary TB context. However, several studies have demonstrated that nicotine, a major constituent of cigarette smoke, worsens disease outcomes through nAChR stimulation (15–17). Additionally, receptors involved in the PPIR are implicated in the pathogenesis of active TB (18, 19). Consequently, the purpose of our study was to determine if the cholinergic system is upregulated during pulmonary TB using an *in vivo* experimental model (20).

We found upregulation of the lung’s extraneuronal cholinergic system during early infection and an even greater potentiation during advanced disease. Furthermore, administering nicotinic ACh receptor (nAChR) α7 and α4β2 antagonists reduced bacterial counts and presented synergism with second-line antibiotics. Finally, we demonstrated that *Mtb* produces ACh and that its growth is potentiated with nanomolar quantities of ACh and inhibited with nAChR antagonists.

## Materials and Methods

### Mice

Pathogen-free, 6–8-week-old male BALB/c mice (aprox. weight 22g), were obtained from the animal facilities of the Salvador Zubirán National Institute of Medical Sciences and Nutrition (INCMNSZ). All work was done according to the guidelines of the Mexican Constitution law NOM 062–200-1999, and approval of the ethical committee for animal experimentation of the INCMNSZ under governmental permit 224. Animals were monitored daily and euthanasia with pentobarbital (Nembutal, 400 mg/kg) was carried out before sample obtainment, as well as on any animal that presented signs of respiratory insufficiency, accentuated cachexia or full immobilization. Mice euthanized for sample obtainment underwent exsanguination by bleeding out the axillary vein. All mouse and sample protocols were carried out in biosafety level III cabinets.

### Induction of Experimental Pulmonary Tuberculosis

Bacterial cultures of the reference strain H37Rv and the multidrug resistant (MDR) isolate CIBIN-99 were prepared as previously described (19, 20). For infection assays, mid-log phases were used. Experimental pulmonary tuberculosis was induced as previously described (20, 21). Mice were anesthetized briefly before inoculation in acrylic lidded boxes through inhalation of 2% sevoflurane vapor (Abbott Laboratories, IL, USA) in 2 L of O_2._ Mice were afterwards immobilized on a unicel cardboard covered by aluminum and infected by the non-invasive instillation (*via* the oro-tracheal route using a rigid stainless steel cannula [Thomas Scientific, Swedesboro, NJ] connected to an insulin syringe) of 2.5 × 10^5^ H37Rv *Mtb* or 3.25 × 10^4^ MDR *Mtb* live bacilli resuspended in 100 µl of sterile isotonic saline solution. Mice were grouped into five-mouse cages that were fitted with microisolators and connected to negative pressurizers within an animal biosafety level III facility.

### Quantification of Lung Acetylcholine

Lungs from infected mice were obtained at 1, 3, 7, 14, 21, 28, and 60 days after infection with *Mtb* and stored at -80°C. Acetylcholine and choline concentrations in lung homogenates were measured by cation exchange HPLC-EC detection as described by Potter et al. (22). Briefly, an analytic column for ACh and choline (MF-6150; Bioanalytical Systems, West Lafayette, IN) and an immobilized enzyme reactor (Bioanalytical Systems) attached in tandem were coupled to the HPLC (model 9012; Varian, Walnut Creek, CA) and connected to the electrochemical detector (Coulochem II; ESA, Chelmsford, MA). For this technique, the isocratic mobile phase (50 mM Tris/NaClO_4_ plus 1% ProClin reagent, pH 8.5) was pumped at a rate of 1 ml/min. Standard curves for ACh and choline (1–100 nM) were used for calibration. The system’s detection limit was ∼0.1 nM for both molecules using 15-μl of the sample. Data were stored and analyzed using a data acquisition and analysis software (Star Chromatography Workstation v4.01, Varian). Total ACh was obtained by adding ACh and choline concentrations and adjusted to protein content (Bradford’s method) and expressed as µmol/mg protein.

### Immunohistochemistry

After removing the right lung for HPLC analysis, the left lungs of BALB/c mice were endotracheally perfused with absolute ethanol and embedded into paraffin blocks. Immunohistochemistry triplicate sections 5 μm thick belonging to three different mice per day of the infection kinetic were mounted on silane-coated slides, deparaffinized and rehydrated. Heat-induced epitope retrieval was carried out using 0.01 mol/L citrate buffer (pH 6.2) and immersing the slides for 10min into a 95°C water bath. The endogenous peroxidase was quenched using a rabbit polydetector peroxidase blocker (Bio SB). Slides were incubated overnight with 200 µl of rabbit anti-mouse polyclonal antibody [ChAT (Santa Cruz, sc-20672 1:250 dilution), α7 nAChR (Santa Cruz, sc-5544, 1:100 dilution). Afterwards, the slides were incubated for 30 mins with mouse/rabbit immunodetector biotin link and rabbit polydetector HRP Label (Bio SB) and bound antibodies were detected with the Rabbit Polydetector DAB kit (Bio SB, Santa Barbara California).

### Treatment Administration

Mice infected with *Mtb* H37Rv were separated into three groups receiving treatment three times per week (Monday, Wednesday, and Friday). The first group received intragastric administration of the α7 nAChR antagonist methyllycaconitine (MLA, Sigma Aldrich - 3 mg/kg) (23). The second group received endotracheal administration, under anesthesia with sevoflurane, of the α4β2 nAChR antagonist dihydro-beta-erythroidine (24) (DHβE, Tocris Bioscience—0.19 mg/kg). The intratracheal dose determination of DHβE was obtained using Akhila et al’s method (25). The third group received 100 µl of saline solution (vehicle) and served as the control group. The two nAChR antagonist treatment groups used for H37Rv infected mice were repeated for MDR *Mtb* infected mice.

To determine if the nAChR antagonists could have synergy with antibiotic treatment, MDR infected mice were additionally treated with an adjusted WHO-recommended regimen consisting of: 1.1 mg/kg of amikacin (A, Sigma Aldrich), 0.55 mg/kg of ethionamide (Et, Sigma Aldrich), 1.1 mg/kg of moxifloxacin (M, Bayer) and 1.65 mg/kg of pyrazinamide (Z, Sigma Aldrich) (26). AEtMZ was administered daily for 5 days per week (Monday–Friday) and served as the exclusive treatment of the control group of MDR TB treated mice. All pharmacological treatments received by mice were suspended in 100 µl of sterile isotonic saline solution, prepared on a weekly basis and stored at 4°C. Treatment schedules began 60 days after infection and continued for a 60-day period. Two independent experiments were performed.

### Measuring Colony-Forming Units

Groups of four animals were euthanized 30 and 60 days after treatment initiation (see section above). In addition, groups of 4 mice infected with MDR *Mtb* were euthanized 7 and 14 days after commencing treatment. Following hilar lymph node and thymic tissue removal, right lungs were frozen and kept at −70°C for the subsequent measurement of CFUs as previously described (27, 28). In brief, lungs were exposed to 40-second cycles in a FastPrep homogenizer (MP biomedicals) within sterile tubes containing 1ml of isotonic saline solution (following the manufacturer’s recommendations). Four serial 10-fold dilutions of each homogenate were spread onto duplicate plates containing Bacto Middle brook 7H10 agar (Difco Labs, Detroit MI, USA) enriched with oleic acid, albumin, catalase, and dextrose; CFU counting was done after a 21-day incubation period.

### Morphometric Analysis

After removing the right lungs for CFU determination, the left lungs of three or four mice from each treatment group from two independent experiments were fixed with alcohol perfused through the intratracheal route. Sagittal lung sections were prepared for histological analysis and stained with hematoxylin and eosin. To determine pneumonic area percentage, each slide was photographed using a camera system (Olympus DP70, Milton Keynes) that obtained an image of the complete lung section, which corresponded to 100% of the lung area. Subsequently, pneumonic areas (foci of consolidation of leukocyte-rich infiltrate in airway walls and adjacent alveolar spaces) were delimited and quantified using an automated histology system (Q-Win Leica 500). Finally, the percentage of the lung surface area affected by pneumonia, was determined. Measurements were carried out blinded with respect to the experimental treatment to which each slide belonged.

### Gene Expression of Lung Proinflammatory Molecules

These assays were performed as previously described (28). Briefly, after removing the right lungs for CFU determination, the left lungs of three or four mice from each treatment group were obtained from two independent experiments. Samples from the evaluated mice were stored in 1.5ml cryotubes containing 1ml of RLT plus, frozen immediately in liquid nitrogen and stored later at -80 degrees centigrade until processing. Each sample was homogenized with zirconia and flint beads (MP Biomedicals) in the FastPrep-24TM equipment for three cycles of 20 s. The RNA extraction was carried out using the commercial RNeasy Mini (Qiagen) kit, following the manufacturer’s instructions. RNA concentration and purity were determined by spectrophotometry (EPOCH 2 spectrophotometer, A260/280). Subsequently, 100 ng of RNA from each lung were used for cDNA production by reverse transcription following the indications of the commercial OmniScript kit (Qiagen). From the complementary DNA (cDNA) obtained for each sample, real-time reverse transcription semi-quantitative PCR (RT-qPCR) was performed with the PCR-RT 7500 instrument (Applied Biosystems) and the QuantiTect SYBR Green Mastermix commercial kit (Qiagen). The expression of iNOS, TNF-α, IFN-γ, and IL-17A transcripts was determined, whose previously reported sequence is specified in **Supplementary Table 1**. The results were standardized with respect to the mRNA content of the β-actin housekeeping gene (whose primer sequence is also specified in **Supplementary Table 1**) of each sample. The appropriate standard curve was included in the individual gene detection, in addition to reverse transcription negative controls. The cycle conditions used were as follows: initial denaturation at 95°C for 15min, followed by 40 cycles at 95 degrees for 20 s, 60 degrees for 20 s, and 72 degrees for 34 s. Data analysis was calculated according to the rate of change in gene expression using the equation described by Livak and the method of 2-ΔΔCT occupying a minimum limit of 35 cycles of TC detection (29).

### Minimum Growth and Inhibitory Concentration Assays

These assays were performed as previously described (28). Briefly, 3 x 10^5^ CFUs (for the ACh assay) or 6 x 10^5^ CFUs (for the nAChR antagonist assay) of the *Mtb* strain H37 Rv were placed in 100 μl of 7H9-OADC supplemented growth media in each well of a 96-well plate. Progressive concentrations of ACh [similar to those reported in human lungs (30)] or nAChR antagonists [in the range of the reported pharmacological dose (23, 24)] diluted in 100 µl of 7H9 medium were subsequently added. A well triplicate with bacteria in 7H9 medium without compounds was used as a positive (bacterial) control and a second triplicate without bacteria was used as a negative (medium) control. In addition, isoniazid (INH) at its minimum inhibitory concentration (0.5 µg/ml) and a solvent control containing saline solution and 7H9 medium were added. Plates were then placed in a humidified incubator at 37˚C with 5% CO2 for 7 days. Four hours prior to the end of the exposure period, MTS (Owen’s reagent, 20 μl/well) was added and its conversion to formazan was tracked every hour spectrophotometrically at 492 nm (BioTek Instruments, ELX 800, USA) and used as an indirect measure of bacterial quantity. The number of bacteria was confirmed counting CFUs taken from 30 µl of the control wells, in addition to the wells exposed to the maximum and minimum concentrations of ACh and conducting four serial 10-fold dilutions into 7H-10 agar plates.

### Macrophage Phagocytosis Assay

The assays were realized as previously described (27). Briefly, murine BALB/c alveolar macrophages (MHS cell line, ATCC CRL-2019) were incubated for 2h in duplicate wells (6 x 10^4^ cells per well) containing 200 µl of RPMI medium supplemented with Fetal Bovine Serum within a 96-well plate with no *Mtb* strains (negative control) or at a multiplicity of infection (MOI) of 5:1 with the following *Mtb* strains (31): H37 Ra (non-virulent strain), 5186 (hypervirulent strain), live and heat-inactivated H37Rv strain. Subsequently, the supernatants were removed and cells were washed 3 times with Fetal Bovine Serum free RPMI supplemented with 1% streptomycin and once with PBS cell solution and were later incubated in 200 µl of Fetal Bovine Serum free RPMI for 2h. The well contents were then collected and MH-S cells were lysed through pipetting, after having added the assay buffer and were later centrifuged at 25,830 x g for 2 mins at 4°C to retrieve supernatants. The supernatants were later collected and kept on ice and subsequent CFU analysis was carried out as a control measure.

### Determination of Acetylcholine in Mycobacteria Growth Culture Medium and in Macrophage Supernatants by Colorimetric Analysis

For colorimetric assays, 3 x 10^5^ CFUs of freshly grown bacterial cultures were obtained, 2, 10, and 18 days after culture initiation (lag, log and stationary growth phases respectively). Each culture sample was diluted in 600 µl of 7H9 medium, centrifuged at 25,830 x g for 2min at 4°C and the bacterial supernatants collected and kept on ice. The remaining bacterial pellet was complemented with 500 µl of assay buffer (Colorimetric Acetylcholine Assay kit, Abcam, ab65345) and exposed to three 20-s cycles at 5500 RPMs in the Precellys 24 tissue homogenizer using the bacterial lysing CK01 kit (Bertin Instruments, France). The samples were then centrifuged at 25,830 x g for 2min at 4°C and the bacterial lysate supernatants collected and kept on ice. Standards, choline probe, acetylcholinesterase solution and the enzyme mix were prepared according to the manufacturer’s instructions of the colorimetric acetylcholine assay kit (Abcam, ab65345). Duplicate wells containing 50 µl of standard curve, reaction mix (background wells), bacterial, and cell samples were prepared with and without acetylcholinesterase to determine total and free choline values respectively. Afterwards, 50 µl of the reaction mix was added to each well and the plate was left incubating at RT for 30min protected from light. A colorimetric reading at OD 570 nm was subsequently made and values were extrapolated from the standard curve. Acetylcholine concentrations were obtained by subtracting free choline from total choline values. In addition, a medium control consisting of 7H9 media without bacterial or cell samples was added and its colorimetric value was subtracted from the value of each sample.

### Statistical Analysis

**Figure 1:**
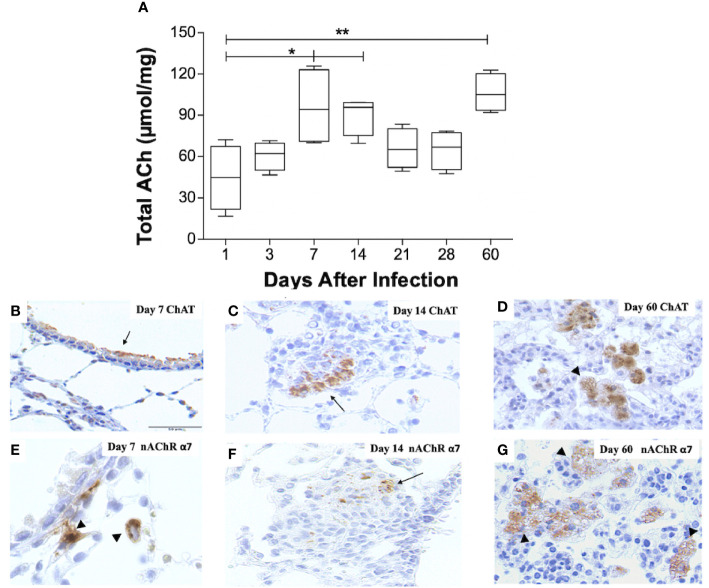
Kinetics of ACh, ChAT, and the α7 nAChR subunit during pulmonary TB. Kinetics of ACh quantification determined by HPLC in BALB/C mice lung pairs infected with the Mtb strain H37Rv (box-and-whisker plots of ACh values). **(A)** Horizontal black lines within the boxes represent the median, while the lower and upper boundaries represent the 25th and 75th percentile, respectively. The upper and lower whiskers correspondingly outspread from the box toward the maximum and minimum values (n = 4 mice per time point/group). One-way ANOVA was used for significance testing, with Bonferroni's post-test for comparisons using lungs of mice from day 1 as the control group (*P < 0.05, **p < 0.01). Representative immunohistochemistry micrographs detecting ChAT and the nAChR α7 subunit **(B–G)**. Representative sections are shown by a 40x objective; bar, 50 um. At day 7 postinfection, there is mild ChAT immunostaining in the bronchial epithelium (arrow) **(B)**. Two weeks after infection, organized nodules constituted by inflammatory cells that correspond to granulomas showed some cells that exhibit ChAT immunostaining (arrow). **(C)**. After 60 days of infection, occasional macrophages with cytoplasmic vacuoles located in pneumonic areas show strong ChAT immunostaining (arrowheads) **(D)**. After one week of infection, some macrophages exhibit intense immunolabeling (arrowhead) of the α7 nAChR subunit **(E)**. Two weeks after infection, some cells in a granuloma show α7 nAChR immunostaining (arrow) **(F)**. After 60 days of infection, numerous vacuolated macrophages and lymphocytes located in pneumonic patches exhibit α7 nAChR immunostaining (arrowheads) **(G)**.

**Figure 2:**
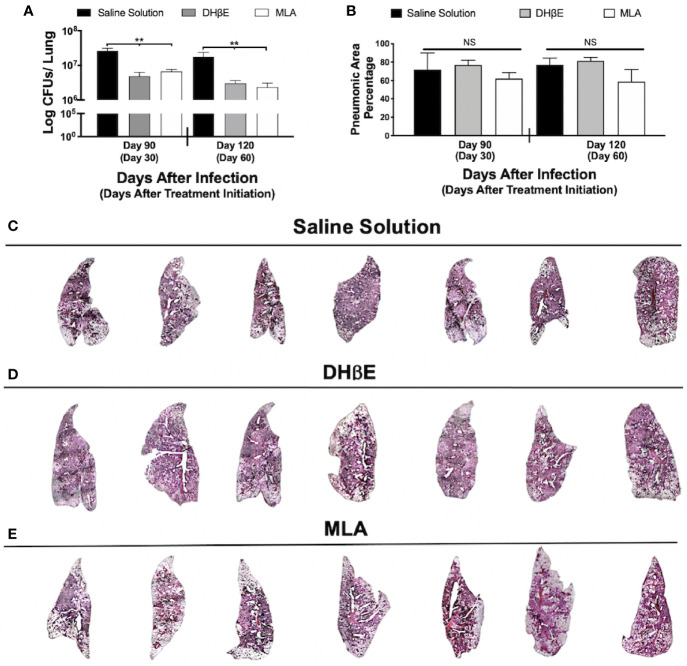
Administration of nAChR antagonists during late infection with Mtb H37Rv reduces lung bacillary burden but does not reduce pneumonia. **(A)** After 60 days of infection with Mtb strain H37Rv BALB/c mice received saline solution (control mice, black bars) or a nAChR antagonist (dihydro-beta-erythroidine [DHβE] gray bars or methyllycaconitine [MLA] white bars). After 30 and 60 days of treatment initiation, quantification of colony forming units (CFU) determined bacilli load in the lungs. **(B)** Percentage of pneumonic areas of the mice infected lungs determined by automated morphometry. Automatized reconstruction of infected lungs after 60 days of treatment with saline solution **(C)**, DHβE **(D)**, or MLA **(E)**. Graphs represent pooled data from two experiments (N = 7 mice) and individual groups display median + interquartile range at each time-point. Significance testing was done using the Kruskal-Wallis test with Dunn's multiple comparison. NS refers to non-significant difference between groups (**P < 0.01).

**Figure 3:**
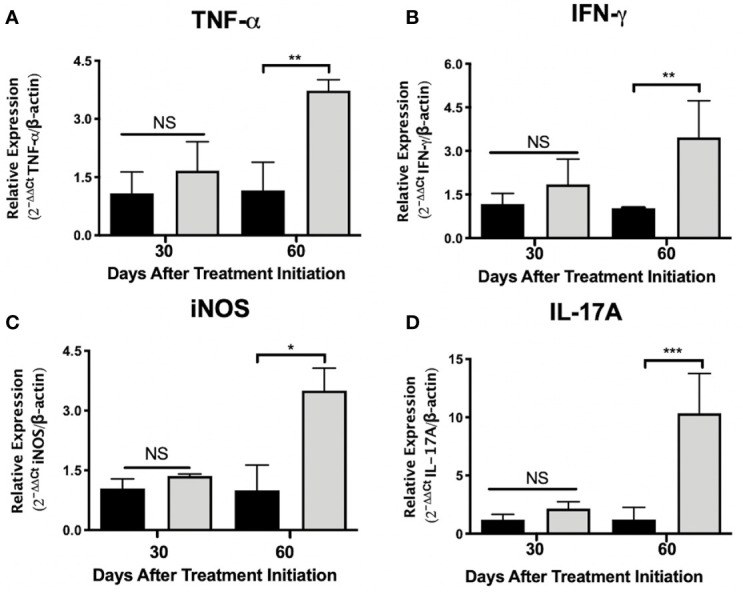
Administration of nAChR antagonists during late tuberculosis infection increases the mRNA transcripts of proinflammatory mediators in the lungs. After 60 days of infection with the *Mtb* strain H37Rv, groups of BALB/c mice were treated with saline solution (control mice, black bars) or dihydro-beta-erythroidine (DHβE, a nAChR antagonist, gray bars). After 90 and 120 days of infection, the indicated gene expression of the proinflammatory molecules in the lungs was quantified by RT-PCR using the 2^ΔΔCT^method in relation to the expression of the β-actin housekeeping gene: TNG-α **(A)** IFN-γ **(B)** iNOS **(C)** and IL-17A **(D)**. Graphs represent pooled data from two experiments (N = 7 mice) and individual groups display mean + interquartile range at each time-point. Significance testing was done using the Kruskal-Wallis test with Dunn’s multiple comparison. NS refers to a non-significant difference between groups (*P < 0.05, **P < 0.01, *** P < 0.001).

**Figure 4:**
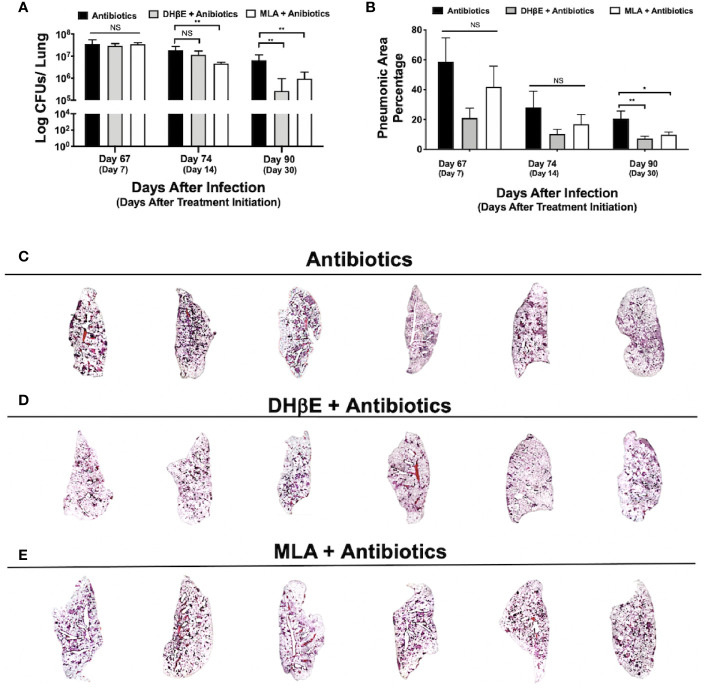
Administration of nAChR antagonists synergizes with antibiotic therapy reducing bacillary burden and pneumonia. **(A)** Lung colony forming unit (CFU) determination in mice receiving the indicated treatments for 7, 14 and 30 days, following 60 days of infection with an MDR strain. **(B)** Percentage of lung area affected by pneumonia determined by automated morphometry. Graphs represent pooled data from two experiments (N = 6 mice) and individual groups display median + interquartile range at each time-point. Significance testing was done using the Kruskal-Wallis test with Dunn's multiple comparison. NS refers to a non-significant difference between groups (*P < 0.05, **P < 0.01). Lower panel **(C–E)** shows low power micrographs of the lungs after 90 days of the indicated treatment.

**Figure 5:**
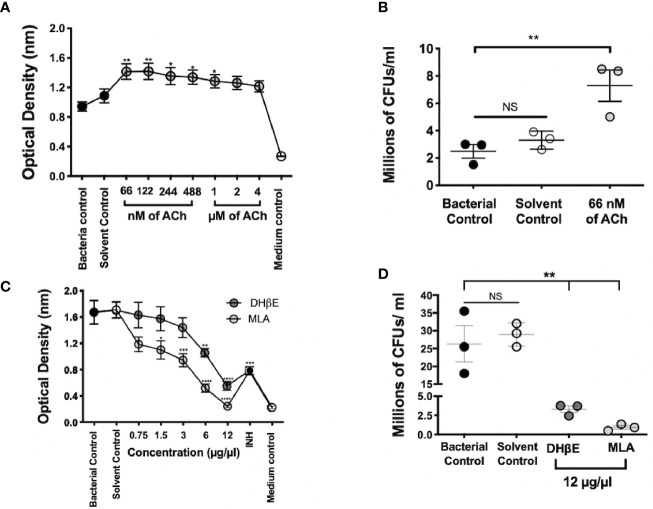
Mtb growth is stimulated by ACh and is blocked by nAChR antagonists. Optical density served as a surrogate of the quantity of live bacteria after the addition of Owen's reagent and was confirmed by CFU counting. The optical density of 3 × 10^5^ CFUs of the Mtb strain H37Rv in liquid culture increases after incubation with nanomolar and micromolar concentrations of ACh (gray circles). Bacteria exposed to nM concentrations and 1 µM concentrations of ACh showed a significant increase in optical density compared with bacterial and solvent controls, indicating bacterial proliferation **(A)**. Increase in mycobacterial CFU burden in bacteria incubated with the ACh concentration that presented the highest optical density (66 nM) compared with bacterial and solvent controls was confirmed through the determination and counting of CFUs **(B)**. Conversely, the incubation of 6 × 10^5^ CFUs of the Mtb strain H37Rv after the addition of incremental concentrations of nAChR antagonists (gray circles) reduced their optical density **(C)** and CFU burden **(D)** compared with bacterial and solvent controls. Data expressed as the mean SEM of three wells and are representative of three independent experiments. ANOVA P < 0.001. Bonferroni's multiple comparison of values from the bacterial control (black circles), which was not exposed to any compound, with bacteria exposed to the solvent control (saline solution), isoniazid (INH), as well as different concentrations of ACh **(A)** or nicotinic antagonists **(B)** is shown (*P < 0.05, **P < 0.01 ***P < 0.001 ****P < 0.0001), NS refers to a non-significant difference between groups.

**Figure 6:**
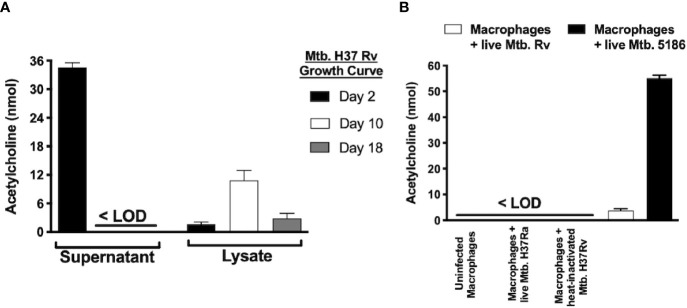
Mycobacterium tuberculosis produces different acetylcholine concentrations during its growth curve and induces acetylcholine secretion in MH-S cells as measured in supernatants and lysates of Mtb H37Rv recovered during three different growth phases. Acetylcholine was detected in Mtb supernatants exclusively during its lag phase and was present in bacterial lysates during all three growth phases but predominated during the logarithmic phase (day 10). **(A)** Acetylcholine concentrations in supernatants of MH-S cells. Acetylcholine concentrations were below the limit of detection (<LOD) in the supernatants of uninfected MH-S cells and in supernatants of MH-S cells infected with avirulent or heat-inactivated Mtb strains. In contrast, infection with live Mtb virulent strains generated detectable acetylcholine concentrations in MH-S cell supernatants **(B)**. Data expressed as the mean + SEM of three wells and are representative of two independent experiments using 3 x 10^5^ CFUs of Mtb. ANOVA P < 0.001. The limit of detection of acetylcholine for the assay was 3 nmol.

The data represented in **Figures 1** and **5**, **6** were analyzed using one way analysis of variance (ANOVA) followed by a Bonferroni correction for multiple comparisons and presented as either the median ± interquartile range or as the mean ± standard error of the mean (SEM) respectively. The data represented in **Figures 2**–**4** were analyzed using the Kruskal-Wallis test with Dunn’s multiple comparison and presented as the median + interquartile range. Tests were performed using GraphPad Prism Software, Inc. (version 8.0, La Jolla, USA).

## Results

### Lung Acetylcholine Increases During Pulmonary Tuberculosis

To record pulmonary ACh concentrations during experimental TB, mouse lungs from representative days of the experimental kinetic underwent HPLC analysis (**Figure 1A**). ACh concentrations obtained during the first day after infection had a mean value of 45 µmol/mg, the lowest in all the kinetic. This mean value, therefore, served as a control to compare against other days. Comparison analysis revealed a two-fold increase (P ≤ 0.05) in lung ACh concentrations 7 and 14 days after infection, which averaged a mean value of 93 µmol/mg. The highest ACh concentration (P ≤ 0.01) was seen after 60 days of infection (106 µmol/mg). Thus, ACh concentrations were measurable during all the TB infection kinetic and showed two peaks, the first at early infection (days 7 and 14) and the highest concentration during late progressive disease (day 60).

### Cholinergic Elements Are Upregulated During Disease Progression

To establish the expression of cholinergic elements (ChAT and α7 nAChR), during early (days 7 and 14) and late (day 60) infection with elevated ACh concentrations, lung tissue sections were studied by immunohistochemistry (**Figure 1**). Mice that had not received intratracheal inoculation of *Mtb* served as controls. Immunohistochemistry analysis of non-infected mice did not show ChAT or α7 nAChR staining (results not shown). However, bronchial epithelial cells and occasional scattered lung fibroblasts and alveolar macrophages in infected mice 7 days after infection display intense ChAT immune staining (**Figure 1B**). After 2 weeks of infection, the airway epithelium appears negative to ChAT immunostaining, while scattered macrophages and macrophages in diffuse inflammatory infiltrates exhibit strong ChAT staining (**Figure 1C**). At these time points, macrophages show intracellular α7 nAChR staining (**Figures 1E, F**). During late disease (day 60 after infection), diffuse inflammatory infiltrates and particularly pneumonic patches reveal strong and widespread ChAT immunostaining (**Figure 1D**), while lung lymphocytes and macrophages express α7 nAChR immunostaining (**Figure 1G**).

### Administration of nAChR Antagonists During Late Tuberculosis Reduces Lung Bacillary Burden but Not Inflammation

To gain insight into the impact of nAChR stimulation by ACh on bacterial burden and inflammation during pulmonary tuberculosis, mice received the α7 nAChR antagonist (MLA) and the α4β2 nAChR antagonist (DHβE) 60 days after infection. Compared with the control group, which only received vehicle treatment, MLA and DHβE administration produced a significant reduction in lung CFUs (**Figure 2A**) but not lung inflammation (**Figures 2B–E**).

### Th1 and Th17 Associated Lung mRNA Transcripts Are Increased After the Administration of a nAChR Antagonist During Advanced Disease

To gain insight into the immunomodulatory effects generated by administering nAChR antagonists during pulmonary tuberculosis, mice were treated with the α4β2 antagonist DHβE, 60 days after infection. Compared with the control group, lung molecular transcripts of the Th1 cytokines TNF-α and IFN-γ and of inducible nitric oxide synthetase (iNOS), increased by more than two-fold, 60 days after the start of treatment (**Figures 3A–C**). Furthermore, administering the nAChR antagonist potentiated the transcription of IL-17A mRNA in the lungs, by approximately five times (P ≤ 0.001) during the same time period (**Figure 3D**).

### Blockade of Nicotinic Receptors During Late Tuberculosis Infection Synergizes With Antibiotic Therapy in MDR Infection by Reducing Lung Bacillary Burden and Lung Inflammation

To determine if nAChR antagonists could be useful in shortening the duration of second-line chemotherapy, 60 days after mice were infected with an MDR TB strain, treatment with second-line antibiotics alone or in combination with MLA and DHβE was initiated. After 14 days of treatment with MLA and antibiotics, a reduction in bacillary burden was observed compared with mice that received only antibiotics (P ≤ 0.01, **Figure 4A**). Moreover, after 1 month, combined treatment with both nAChR antagonists and antibiotics produced a significant reduction in bacillary burden compared with mice that received only antibiotics (P ≤ 0.01, **Figure 4A**) and additionally reduced pulmonary inflammation (**Figures 4B–E**).

### Acetylcholine Stimulates *Mtb* Growth, While nAChR Antagonists Possess Bactericidal Activity

Mycobacterial incubation with ACh or nAChR antagonists determined the effect of bacterial growth to cholinergic agonism and antagonism; and this was assessed through colorimetric detection and CFU count. Nanomolar and micromolar concentrations of ACh increased optical density (P ≤ 0.01 and P ≤ 0.05 respectively, **Figure 5A**) and bacterial CFU numbers (**Figure 5B**). Conversely, the addition of incremental concentrations of nAChR antagonists reduced optical density (**Figure 5C**) and bacterial CFUs (**Figure 5D**). Thus, suggesting that ACh may function as a mycobacterial growth factor.

### Diverse Acetylcholine Concentrations Are Present in *Mtb* Supernatants and Lysates During Its Growth Curve and in Supernatants of Infected Alveolar Macrophages

To determine if *Mtb* is capable of ACh production, supernatants and bacterial lysates of the *Mtb* strain H37Rv after 2, 10, and 18 days of culture initiation that corresponded to the lag, log, and stationary growth phases, respectively underwent ACh measurement using a colorimetric assay (**Figure 6A**). ACh was detected in supernatants (~35 nM) only during *Mtb*’s lag phase and in lysates predominantly during the logarithmic phase (~10 nM). Additionally, when murine alveolar macrophages of the MH-S cell line were infected with virulent (H37Rv and 5186) or avirulent (H37Ra) strains of *Mtb*, the virulent, but not the avirulent or heat-inactivated Mtb strains, induced ACh secretion in culture supernatants (**Figure 6B**).

## Discussion

The cholinergic system is present within the lungs where it aids in maintaining their proper physiological functioning. Altered lung cholinergic expression typically occurs in chronic obstructive pulmonary disease (COPD) and results in pathological processes such as mucus hypersecretion and fibrosis (32, 33). Although alteration of the lung’s cholinergic system has not previously been demonstrated in advanced pulmonary TB, pathological manifestations similar to those occurring in COPD are characteristically observed (33). Larcombe and colleagues previously demonstrated that basal cholinergic tone is completely absent in BALB/c mice (34). However, using a well-characterized BALB/c mouse TB model (20), we have shown for the first time that upregulation of the lung’s non-neuronal cholinergic system occurs during experimental pulmonary TB. Our results suggest that cholinergic upregulation is generated as a consequence of *Mtb* infection favoring immunopathological progression and extensive lung inflammation partly due to the suppression of protective Th1 and Th17 immune responses.

Acetylcholine has generally been regarded as a classical neurotransmitter, despite the fact that it was first identified in the spleen, an immune organ (35). Currently, the capacity to synthesize ACh has been detected in both neuronal and non-neuronal cells, which release it in a rapid or slow fashion respectively (10, 36). ACh has been termed the ‘universal cytotransmitter’ in reference to its extensive expression (35) and almost every lung and airway cell is either a source or a potential cholinergic target. In fact, a myriad actions are regulated by ACh through stimulation of muscarinic and nicotinic ACh receptors (mAChRs and nAChRs), including bronchial epithelial cell growth, goblet cell secretion and regulation of mucosal surface barrier function (4, 37).

In our experimental model, we have observed substantial concentrations of lung ACh during the complete course of infection. Increased ACh concentrations in the lungs and other organs secondary to intracellular pathogen exposure have previously been described (38, 39). For example, the parainfluenza virus potentiates lung ACh concentrations through mRNA degradation and cleavage of sialic acid residues of the inhibitory mAChR2 which regulates both neuronal (38), and non-neuronal ACh secretion (38, 40). Although we did not measure mAChR2 expression, previous reports suggest that *Mtb* is capable of altering G-protein-coupled receptor expression during infection (41). Alternatively, cytokines such as TNF-α and IFN-γ which are induced under LPS challenge (39), and are also highly induced in our model (20) should be able to increase ACh availability through mechanisms such as the inhibition of acetylcholinesterase, the ACh degrading enzyme (38, 39). Finally, neurohormonal changes described in our model (27, 42–44) may also be responsible for potentiating lung ACh concentrations through non-neuronal ACh release during infection. This could occur through reported mechanisms which include the inhibition of organic cation transporters by steroids and noradrenaline (2, 37) and/or the stimulation of β2-adrenergic and cholecystokinin receptors (45). Importantly, these neurochemicals and their receptors are present in mammal lungs (42, 46). Our model has restrictive parameters including mouse strain, sex, and age, which may bias cholinergic expression and thus might not be representative of the cholinergic dynamics of human disease. However, the fact that pulmonary ACh concentrations vary during disease progression suggests that the lung cholinergic system might carry out different roles during early and late TB.

Immune cells have the capacity to synthesize, release, and respond to molecules classically classified as neurotransmitters (10). Indeed, CD4+ T lymphocytes are important non-neuronal sources of ACh (1), although almost all cell types within the lungs express ACh synthesizing enzymes (2, 47). Here we have documented the upregulation of ChAT, the main ACh synthesizing enzyme in peripheral tissues and non-neuronal cells (1, 2), during *in vivo* TB infection. Upregulation of ChAT during early disease occurs in lung epithelial cells, which are known to be directly devoid of neuronal ACh (4). While during advanced disease, ChAT upregulation occurs to an even greater extent within immune cells, particularly lymphocytes and macrophages. Moreover, our *in vivo* and *in vitro* results suggest that *Mtb* infection induces ACh production. Interestingly, when comparing the levels of ACh detected during infection of macrophages of the MH-S cell line with the standard Mtb H37Rv strain (31), ACh detection was either potentiated or nullified when the infection was carried out with the hypervirulent *Mtb* strain 5186 (31) or the hypovirulent Mtb strain H37Ra (15) respectively. This would suggest that the degree of virulence of the strain of mycobacteria correlates with the levels of ACh production induced during infection. Furthermore, heat-inactivation of the H37Rv strain also nullified ACh detection, suggesting that ACh production requires intact bacteria. It is therefore plausible that the bacilli might have either produced the measured ACh or alternatively, induced ACh production by MH-S cells during infection. Heat-killed and avirulent Mtb may therefore lack the biochemical machinery to either produce ACh or induce production of ACh by MH-S cells. Induction of ChAT through T cell receptor (48) and toll-like receptor (12, 39) stimulation has been demonstrated in several immune cell types including T cells, macrophages and dendritic cells (1, 36, 45). Moreover, the induction of ChAT in immune cells through neurohormonal receptor stimulation has also been suggested (45) and is especially relevant, as several neurohormonal alterations have been described in our experimental mouse model (27, 42–44). Importantly, we were unable to experimentally differentiate the source of ACh production during infection and therefore cannot conclude whether the ACh was produced by the mycobacteria, by cells during their infection or by both.

Cholinergic receptors are expressed on several immune cells including macrophages, monocytes and lymphocytes (1, 10, 36). Leukocytes express all nAChR subtypes and these help trigger mechanisms of action in these cells that are different from those triggered in neurons. Instead of forming voltage-activated gates, nAChRs release intracellular calcium stores after sequestering intracellular chains from neighboring receptors which modify second messenger, transcription factors and enzyme activity (1, 36, 49–52). The α7 nAChR is perhaps the most studied nAChR in the regulation of the immune response (10, 12). However, other receptors such as the α4β2, α9 or α10 nAChRs are known regulators (50, 53). Activation of nAChR receptors can alter cytokine production in alveolar macrophages (7, 54). Moreover, nicotine affects the migratory lung response of key leukocyte subpopulations (neutrophils, macrophages and lymphocytes) which are critical in regulating infection (55). This might explain the increased susceptibility of smokers or cultured leukocytes exposed to nicotine, to a long list of infections caused by microorganisms including *Streptococcus pneumoniae, Hemophilus influenza, Chlamydia pneumoniae, Legionella pneumophila, Pseudomonas aeruginosa, and Cryptococcus neoformans* (5–7, 13, 55, 56).

Previously, Matsunaga et al. used non-infected MH-S alveolar macrophages, a BALB/c mouse cell-line, to demonstrate the expression of α4β2 but not α7 nAChR transcripts by RT-PCR. A limitation of our study is that we did not measure other nAChR subunits such the α4β2 nAChR. However, in concordance with Matsunaga et al’s findings, we did not observe the expression of the α7 nAChR subunit in alveolar macrophages of non-infected mice (7). Contrastingly, after *Mtb* infection lung macrophages showed intracellular nAChR α7 subunit expression during early TB and abundant membranal α7 nAChR expression in lymphocytes and during advanced disease in macrophages with foamy appearance present in pneumonic patches. We speculate that this effect could be caused by a product of the infecting bacilli, which could be directly inducing the observed α7 nAChR overexpression. This mechanism has been described in HIV infection, where it was mediated by gp-120 and resulted in the paradoxical potentiation of the inflammatory response (57). An alternative mechanism that could be responsible for nAChR α7 overexpression would be the interference of nAChR expression and function by host steroids (2, 37). The alteration of host steroid production and performance has previously been documented in our model (43, 44).

The previously mentioned study by Matsunaga et al. using a model of *Hemophilus influenza*, reported immunomodulatory effects after general nAChR treatment but not after α7 selective treatment (7). In contrast, an *ex vivo* study of *Mtb* proliferation found that the α7, β2 and β4 nAChR subunits were crucial in regulating the effectiveness of macrophage containment of bacterial proliferation. The same authors concluded that nAChRs directly modulate macrophage autophagy and indirectly modulate Treg TGF-β and IL-10 production (16). While the α7 nAChR has a low affinity for acetylcholine and is rapidly desensitized, the α4β2 nAChR possesses high affinity for acetylcholine and is desensitized slowly (58). In our model, the administration of both α4β2 and α7 nAChRs nAChR antagonists to mice infected with drug susceptible *Mtb* decreased lung CFU burden but not inflammation. This suggests that immunomodulation secondary to cholinergic upregulation of α4β2 and α7 nAChRs might offer protection from excessive inflammation, but at the same time, decrease the proinflammatory protective immune response contributing to disease progression.

In our experimental model, several neurochemicals, their enzymes and receptors present variability in their concentrations during the course of disease. This variability is probably induced by the host or the bacteria, partly to regulate the immune response (27, 42, 43). Here, we have documented varying kinetics of ACh, ChAT and α7 nAChR expression throughout the disease’s progression. During very early disease (days 1 and 3 after infection), we recorded the lowest lung concentrations of ACh and observed ChAT and α7 nAChR expression in bronchial epithelial cells and alveolar macrophages, respectively. This suggests that although ACh concentrations are not yet significantly measurable in a global manner within the lung, the cholinergic system begins to be expressed at a local level in the first cellular responders of the infection, where it might play an immunoregulatory role (15). However, during early disease (days 7 and 14 after infection), ACh concentrations increased significantly and the enzyme ChAT began to be expressed in macrophages and lymphocytes present in diffuse inflammatory infiltrates. Contrastingly, during this time period, the α7 nAChR appeared to be underexpressed in the lungs. We therefore believe that during this stage the transient upregulation of other cholinergic receptors, such as certain muscarinic receptor subtypes, might be taking place to promote protective innate cellular immune responses (47, 54). Further on, during advanced infection (days 21 and 28 after infection), when the Th1 and Th17 adaptive immune responses and M1 macrophages contain bacilli (20), we observed downregulation of ACh expression in the lung. A decrease in cholinergic activity at this stage might be orchestrated by the host to favor the development of a protective adaptive immune response. Contrastingly, during late disease (60 days after infection), when the Th2 and T regulatory responses facilitate bacilli replication and lung pneumonia ensues (20), there is a notable upregulation of lung ACh, ChAT, and α7 nAChR in lung immune cells. It is likely that cholinergic potentiation during advanced disease is induced by the host to downregulate the cellular immune response and avoid overt lung damage. Paradoxically, this strategy ends up favoring bacterial replication and disease progression.

Th1 cytokines, such as TNF-α and IFN-γ, have been classically regarded as correlates of protection during advanced pulmonary tuberculosis (59). In addition, the production of reactive oxygen species within the phagosome represents a crucial mechanism of *Mtb* eradication and is mainly regulated by the enzyme iNOS, which in turn is regulated by Th1 cytokines (60). We observed potentiated lung transcription of iNOS, TNF-α and IFNγ after the administration of the nAChR antagonist DHβE. Regulation of Th1 cytokines and reactive oxygen species by nAChR stimulation has in fact been described in several models (7, 49–51, 61). However, this is the first time that it has been reported in an *in vivo* model of TB. In addition, the administration of DHβE potentiated the transcription of Th17 cytokines in our model. The Th17 response has been associated as a correlate of protection in pulmonary TB and is characteristically represented by the cytokine IL-17A (62). Importantly, the regulation of the Th17 response by nAChRs has been previously described in a different disease model (52). Furthermore, in a recent report, the stimulation of nAChRs after *Mtb* infection of human monocyte-derived macrophages decreased the production of both Th1 and Th17 associated cytokines (IL-6, IL-8, and TNF-α) (17). While in an *ex vivo* TB infection model, nAChR stimulation appeared to potentiate Th2 and T regulatory cytokines, which are known to oppose protective Th1 and Th17 responses (16). In summary, the administration of an α4β2 nicotinic antagonist appears to counteract the immunomodulatory alterations caused by the bacilli, which are responsible for the disease’s progression. A limitation of our study is that we did not measure MLA’s effect on Th1 and Th17 cytokines or the effect of nAChR antagonists on Th2 and T-regulatory cytokines.

Currently, drug-sensitive TB can be cured using combination therapy. However, the therapeutic regimen requires an average intake of four antibiotics for at least 6 months resulting in significant adherence complications. Moreover, a recent meta-analysis concluded that patients that were taking first-line therapy for TB were significantly at risk for developing disease recurrence, as well as multidrug resistance (63). In the past year, approximately half a million cases and more than two hundred thousand deaths were attributed to MDR-TB (14). MDR-TB cases require treatment with second-line therapy, which is significantly more expensive, toxic, and less effective than first-line therapy (64). When given in addition to second-line antibiotic therapy, nAChR antagonists accelerated the reduction of both lung CFU burden and inflammation. Therefore, the administration of nicotinic antagonists could potentially reduce treatment duration if administered with antibiotic therapy, a goal proposed by the WHO (14).

Remarkably, the cholinergic system is phylogenetically ancient and its components have been reported in vertebrate and invertebrate organisms, including insects, plants, fungi, and even bacteria (2, 65). Pathogenic bacteria such as *E. coli* and *S. aureus* are capable of synthesizing ACh using an uncharacterized enzyme (65). Moreover, microorganisms such as T. *kodakaraensis*, an archaebacteria, can synthesize ACh through ChAT activity (66). Cholinergic stimulation has been implicated in bacterial functions such as motility (67), and in growth regulation in plants and mammalian cells (65, 67, 68). Here we report that the addition of ACh potentiates the growth of *Mtb* and we also demonstrate that Mtb is capable of producing ACh *in vitro*. Importantly, the highest quantities of ACh in *Mtb* supernatants were found during its lag phase suggesting that during its initial growth phase, *Mtb* might secrete ACh as an autocrine or paracrine growth factor. Contrastingly, the highest quantities of ACh in *Mtb* lysates were found during the subsequent exponential growth phase. This is consistent with its role as a growth factor, as this phase is associated with the highest bacterial growth and bacterial uptake or internal production of a growth factor would be anticipated to take place at this stage (69). Other secreted growth factors produced by *Mtb* that are active at very low concentrations and which regulate its virulence have in fact already been isolated (70, 71). Therefore, the existence of additional secreted growth factors produced by *Mtb* which impact its virulence remains viable.

Furthermore, previous studies confirmed increased *Mtb* growth when exposed to nicotine supplemented medium (72), which would insinuate that the potentiating and hindering effects of ACh and nAChR antagonists respectively on *Mtb* growth that we have shown might be due to their interaction with an ancestral bacterial nAChR. In fact, two ancestral nAChR members of the pentameric ligand-gated ion channel (pLGIC) family have already been described in other bacteria, the homologous protein from *Gloeobacter violaceus* (GLIC) and *Erwinia chrysanthemi* (ELIC) (73). Predictive phylogenetic analysis found bacterial pLGIC genes in many taxons and importantly they were present in several bacterial pathogens. These pentameric receptors mediate chemo-electric signal transduction and are constituted by homologous subunits and an extracellular domain with five agonist binding sites. Despite homology in sequence and structure, significant phylogenetic divergence exists between prokaryotic and eukaryotic pentameric receptors and their properties and form of functioning in bacteria remain unclear (74).

In conclusion, our findings suggest that cholinergic upregulation during pulmonary TB favors *Mtb* infection not only by altering the protective immune response but also by aiding bacterial proliferation. Further investigations must, therefore, explore the mechanisms that favor upregulation of the cholinergic elements discussed here as well as the role of other cholinergic factors during disease progression.

## Data Availability Statement

The raw data supporting the conclusions of this article will be made available by the authors, without undue reservation.

## Ethics Statement

The animal study was reviewed and approved by Ethical Committee of Animal Experimentation (CICUAL).

## Author Contributions

RH-P and LI-W contributed to the theoretical background and design of the experiments. LI-W performed all of the experiments. LI-W and DG-R performed the data analysis. JB-P performed the Mtb infections and supervised the animal experiments. BM-C performed and supervised the molecular biology, immunohistochemistry, and acetylcholine-colorimetric experiments. DM-S performed and supervised the Mtb culture preparations and minimum inhibitory concentration experiments. JC performed and supervised the HPLC analysis. LB, JLMF and ISP-G performed and supervised the cell-culture experiments. LI-W and RH-P wrote the manuscript. JB-P provided the funds. All authors contributed to the article and approved the submitted version.

## Funding

This work was financed by CONACYT, through the convocation of basic science support CB-2015-01; project # 255209. LI-W is a doctoral student from Programa de Doctorado en Ciencias Biomédicas, Universidad Nacional Autónoma de México (UNAM) and received fellowship 587041 from CONACYT. This manuscript is part of his Ph.D. thesis under the aforementioned program and university.

## Conflict of Interest

The authors declare that the research was conducted in the absence of any commercial or financial relationships that could be construed as a potential conflict of interest.
