# Supplementary figures and images for: The Cholinergic System Contributes to the Immunopathological Progression of Experimental Pulmonary Tuberculosis
Source: Front Immunol. 2021 Feb 18;11:581911. doi: 10.3389/fimmu.2020.581911 (PMC7930380; doi:10.3389/fimmu.2020.581911)

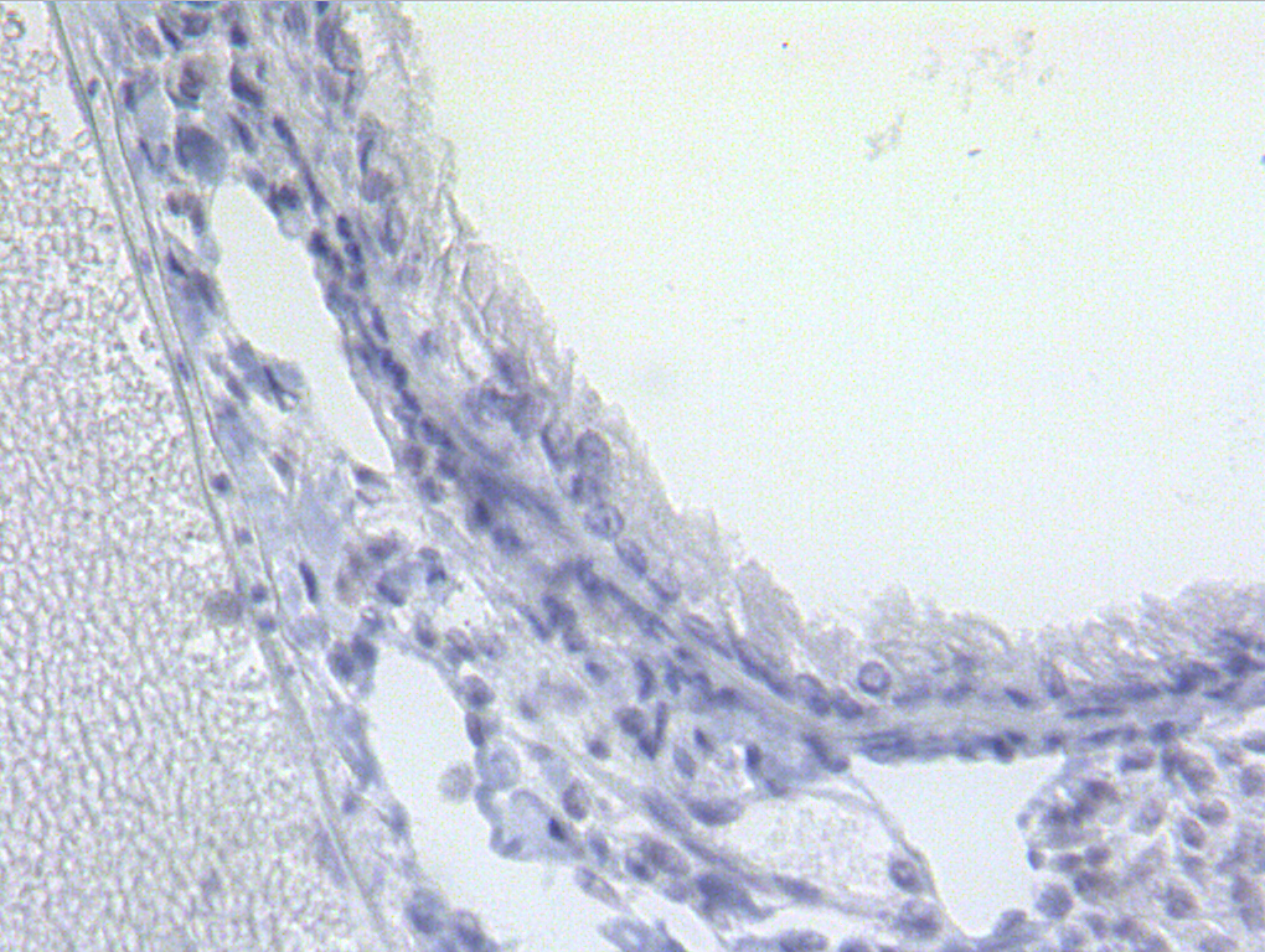

Supplement: Supplementary file 1 [file Image_1.tiff]

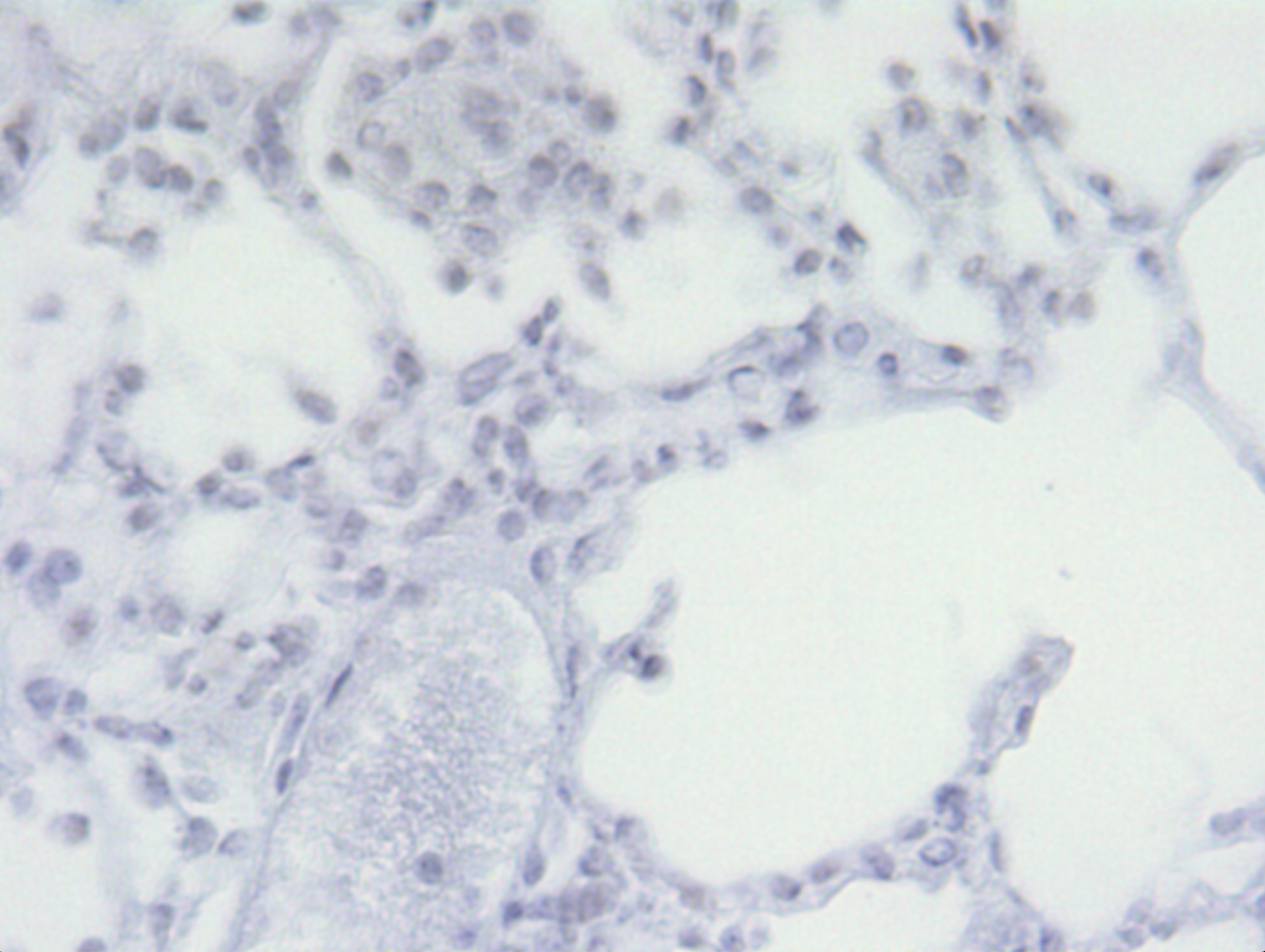

Supplement: Supplementary file 2 [file Image_2.tiff]

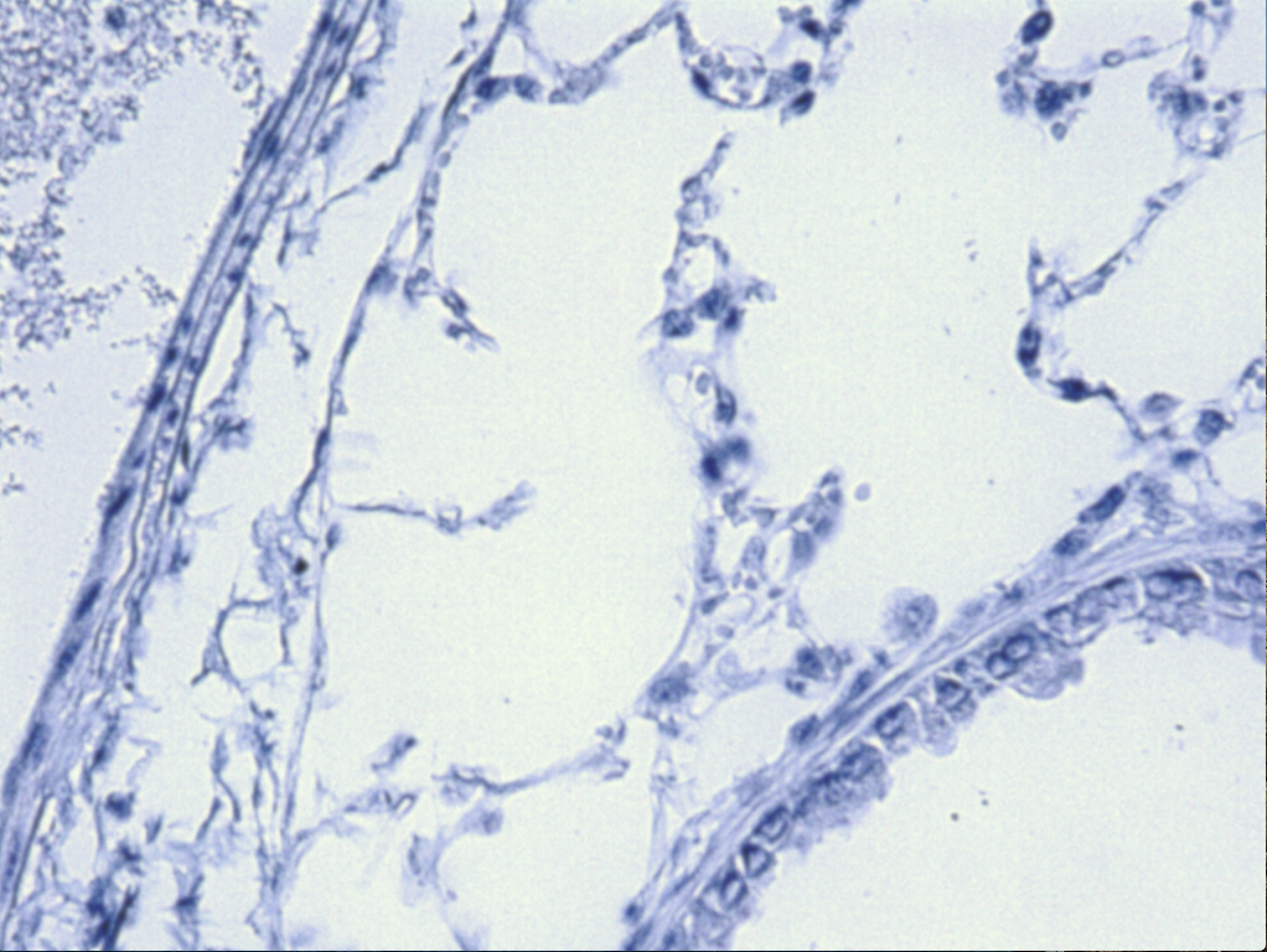

Supplement: Supplementary file 3 [file Image_3.tiff]
