# Supplementary material for: The Cholinergic System Contributes to the Immunopathological Progression of Experimental Pulmonary Tuberculosis
Source: Front Immunol. 2021 Feb 18;11:581911. doi: 10.3389/fimmu.2020.581911 (PMC7930380; doi:10.3389/fimmu.2020.581911)
Supplement: Supplementary file 4 [file Table_1.pdf]

Tabla 1. Forward and Reverse sequences used for Real-Time quantitative PCR analysis.

Gene	Forward Sequence 5'-3'	Reverse Sequence 5'-3'
β-Actin	CTA AGG CCA ACC GTG AAA AGA	ACA ACA CAG CCT GGA TGG CTA
TNF-α	TCG AGT GAC AAG CCT GTA GCC	TTG AGA TCC ATG CCG TTG G
IFN-γ	GGT GAC ATG AAA ATC CTG CAG	CCT CAA ACT TGG CAA TAC TCA TGA
IL-17A	CAC CAG CTG ATC AGG ACG CGC	GGA CCC CAA CAG CTG GAA TAG
iNOS	CAT TTC GCT GTC TCC CCAA	AGC GAG GAG CAG GTG GAAG
